# Supplementary material for: High dropout rate from maternity continuum of care after antenatal care booking and its associated factors among reproductive age women in Ethiopia, Evidence from Demographic and Health Survey 2016
Source: PLoS One. 2020 Jun 12;15(6):e0234741. doi: 10.1371/journal.pone.0234741 (PMC7292400; doi:10.1371/journal.pone.0234741)
Supplement: Attachment — Submitted filename: Response to Reviewers.docx [file pone.0234741.s001.docx]

**Author's response to reviews**

High dropout from maternity continuum of care after antenatal care booking and its associated factors among reproductive age women in Ethiopia, Evidence from Demographic and Health Survey 2016 (PONE-D-20-01331)

# Atalay Goshu Muluneh*^1^, Getahun Molla Kassa^1^, Geta Asrade Alemayehu^2^, Mehari W/Mariam Merid^1^

*^1^Department of Epidemiology and Biostatistics, Institute of Public Health, College of Medicine and Health Sciences, University of Gondar, Gondar, Ethiopia*

*^2^Department of Health System and Policy, Institute of Public Health, College of Medicine and Health Sciences, University of Gondar, Gondar, Ethiopia*

**Corresponding author:*

Atalay Goshu Muluneh

Email addresses:

AG: [goshuatalay12@gmail.com](mailto:goshuatalay12@gmail.com)

GM: [getahunm08@gmail.com](mailto:getahunm08@gmail.com)

GA: [getasrade64@gmail.com](mailto:getasrade64@gmail.com)

MWM: [mehariho19@gmail.com](mailto:mehariho19@gmail.com)

Postal address: P.O. Box 196, Gondar, Ethiopia

Version 1, Date: April, 2020

With regards!

From: Atalay Goshu Muluneh

Correspondence Author

**Author's response to reviews:** Find it below

To: PLOS ONE, editorial office

**Subject:** Submitting a revised version of a manuscript

**Object:** High dropout from maternity continuum of care after antenatal care booking and its associated factors among reproductive age women in Ethiopia, Evidence from Demographic and Health Survey 2016 (PONE-D-20-01331)

We would like to thank the reviewers and editor for sharing the view and experience. The comments are very important that will improve the manuscript. The point-by-point responses for each of the comments and the revised manuscript are provided in the attached documents.

**POINT BY POINT RESPONSES**

We would like to take this opportunity to thank the reviewers and the editor for sharing their view and constructive comments. The point-by-point responses for each of the comments are provided in the following pages.

Authors’ response of reviewer 1

| **REVIEWER 1 COMMENT** | **AUTHORs RESPONSE** |
| --- | --- |
| 1. Abstract The second sentence under “methods” should be deleted. Authors had initiated stated that they conducted secondary data analysis, therefore, the second sentence is not necessary In the results, they should present dropout rate for each indicator of maternal care. This becomes necessary because there were results for factors associated with each of them. | **Thank you very much!**   - Thank you and we update based your recommendation. We add the dropout rate “2,092(44.58%), 2,183 (46.52%) and 4,086(87.07%) dropped from recommended number of ANC, Institutional delivery and postnatal care visit respectively.” See line 44-46. |
| 2. This section lacks coherence. Although, the authors tried to justify the paper by making reference to high level of childhood and maternal mortality, they failed to situate the work within the larger body of knowledge in the subject area. It’s difficult to know what new knowledge is been added o maternal care broadly and specifically in Ethiopia. In the Methods section, authors might want to provide richer contextual information about maternity care in Ethiopia and how it has fared over time. The suggested recommendation should be more specific and linked to the results of the study. | **Thank you for your great insight and we acknowledge the gaps.**   - We try to rearrange - The 1^st^ paragraph of the background is about status of maternal health and continuum of care in Ethiopia. see line 68-84 - We update the recommendation based on specific findings. See line 58-61 |
| 3. The manuscript was not presented in an intelligible fashion and written in standard English | Thank You!  Accepted and it has been given for a local language editor and we update based on his edition.  Here is the professional Experience of the local language editor:  **Name of the Editor**: Demeke W.Ghiorges  **Age**: 72 years  **Academic degrees**: BA in English language and literature, Addis Ababa University, 1983, M.A. teaching English as a foreign language, Addis Ababa university 1991  **Academic rank:** Assistant professor in English  **Teaching experience**: over 30 years  **Editing Experience:** Language editor of the Journal of Medicine and Biomedical Sciences, College of Medicine and Health Sciences, the University Of Gondar, Ethiopia  **Telephone:** 0918034043 |

Reviewer 2

| **REVIEWER COMMENT** | **AUTHORs RESPONSE** |
| --- | --- |
| 1. I feel the title can be more concise. Having read the manuscript, perhaps phrases like “after antenatal care booking” can be left out of the title? | **Thank you for your valuable comment!**   - Accepted:   See the title page line 1 and line 35 of the abstract section |
| 2. Abstract Objective: Please restate objective to be more concise. For example, is it associated factors of continuum of care or associated factors on/along continuum of care? | **Thank you!**   - We update the objectives based on your comment and see line 35 of the abstract |
| 3. Absttract: Methods • I am not clear on the weighting approach, and whether it refers to the original survey or the study being reported. | **Thank you in advance!**   - The survey recommends weighting the data for any inferential statistics. “Due to the non-proportional allocation of the sample to different regions and their urban and rural areas and the possible differences in response rates, a sampling weight must be used in all analyses using the 2016 EDHS data to ensure the actual representative of the survey results at both the national and domain levels(1)” . Weighting the data is a must to balance the above mentioned differences in response rate, and non-proportional allocations. Weighting variables are also available for each data set and we use the recommended weighting variable for women data. See the detail from the main Ethiopian Demographic and health Survey 2016 report Appendix A4. Sampling Weight on page 337. |
| 4. Abstract results, what are the factors associated with completing the whole continuum? Would be interesting to see if there are any significant differences between the non-completed and completed groups. • I feel factors associated with dropout from delivery care could be stated more concisely with some editing • Being informed or LACK of information on pregnancy complications from ANC was associated with ANC, delivery and PNC? | **Thank you,**   - We are interested to find factors of why women dropout from maternity continuum of care. We fit three models for recommended number of ANC visit (Four and above ANC), Institutional delivery and postnatal care. Information given about complications of pregnancy during their ANC visit was significantly associated with all continuums of cares (from recommended ANC, Skilled delivery and PNC) but other variables were specific for all continuums of care. |
| 5. Abstract, Conclusion • Perhaps less repetition of results (The 6.56% figure) and add a sentence on what the study recommends? | **Acknowledged!**   - We update the conclusion based on your recommendation and amended objective. See line 54-59. |
| 6. Line 57-58 maybe a sentence or two to give reader idea of the extent of maternal health challenge in Ethiopia, such as mortality rate and some of the specific challenges faced? A short description of maternity continuum of care as it is conceptualized in this study can be included in the background. | **Thanks,**   - **According to the EDHS 2016 report, Ethiopian women had:**   - Ethiopian women had a 21 per 1,000 women life time risk of death related to pregnancy   - a maternal death of 412 per 100,000 live births - See line 75-77 of the background |
| 7. Line 69-71 I feel that more precise/objective terms can be used here – instead of “lion’s share” perhaps an exact statistic to give reader an idea of the magnitude of the problem. | **Thank you,**   - There are a number of literatures stating the importance of maternal continuum of care to reduce maternal and child mortality. For example 65% of the maternal deaths and 75% of maternal deaths occur in the early postnatal period which could be prevented by proper postnatal care services. In Ethiopia if a timely and comprehensive postnatal care service was given, neonatal mortality could be reduced by 10-17% (2). |
| 8. Methods Line 88 – was the study representative at the national level? The study design section should include information about study design of this particular study, not just the original survey. What is the difference between “7,589 women who give birth within five years before the survey were interviewed for ANC visit and place of birth” and the “4,693 women data that give birth within the survey after antenatal booking”. Is one a sub-sample of another? Is the sample of 4693 also representative nationwide? | **Thank you very much,**   - The data was representative at national level. Samples were taken from all regions including urban, rural and other pastoralist communities with large sample size. The major demographic and Health Survey reported as a standard and representative nationwide survey. We hope the 4,693 women who give birth after ANC booking are still representative. - Sure, the original survey was a cross-sectional study and here we use the control study design approach. See line 109-110 of the methods section - Other single studies try to address factors related to antenatal care booking. As a result, we are interested to determine factors why women lost from completing recommended ANC visit in Ethiopia after 1^st^ ANC booking. So a total of 7,589 women give birth within five years before the survey. Among those, 4,693 of women were booked for ANC. And we use them as a study population. See line 129-134 |
| 9. Results Line 134-136 also please offer a contrast with urban residents – how many attended ANC, institutional delivery etc. | **Thank you!**   - The detail is found in Table 1 as a 1^st^ variable. Please see table 1^st^ row. The table shows the ANC, Institutional delivery and PNC for each variables |
| 10. Results: Line 143 “Among 4,693 women, only 308 (6.56%) or 11.84% from those having four and above ANC visit have had complete maternity continuum of care”. This statement is confusing, I am not sure what the finding is. “among those who give birth from the health institution  - Line 145 after 4 and above ANC visit, 779 (29.96%) dropped from institutional delivery” How do people who gave birth in institution drop out from institutional delivery? | **Thank you!**   - Among women who booked for ANC only 308 (6.56%) used the complete continuum of care. Among women who had a recommended ANC (four and above) 11.84% used the complete continuum of care.   - From the line 145, as you know mothers who had a recommended number of ANC visit (Four and above) are expected to give birth at health institutions but 779(29.96%) of them give birth out of health institution. Those who give birth at the health institution were could not drop from institutional delivery. They already had institutional delivery. |
| 11. Results: Line 144 “Among the women who were booked for ANC, the dropout rate was 10.85%” Drop out from what, antenatal care? Perhaps for precision and to write more concisely, phrases like “4 and above ANC visit” can be shortened to “completed ANC”. It helps when there is a long sentence describing findings, the reader doesn’t get confused In the method the authors described what drop out from the continuum meant for each stage, but not what the dropout rate is? How it is defined. And it should be stated clearly more than one dropout rate is considered by referring to drop out rateS, perhaps even in the title. Please adjust Table 2 to look neater – that is, not cut out short words such as “visit” | **Thank you!**   - 10.85% dropout from complete (recommended ANC i.e. four and above) - The definition for dropout from rate from continuum of care was defined as proportion of women who had completed the recommended ANC among the study participants, women who give birth at the health institution, who had postnatal care visits. |
| 12. Results: Table 2 : It is not clear to me what these proportions are. If drop out from institutional delivery for the category >4 visits is 65%, does that mean those women delivered at home? And how does one conceptualize a 35% drop out from home delivery then? Drop out from home delivery was not even specified as a variable of interest in the methods section– it does not constitute part of the definition of continuum of care in this study. Other variables such as dropout from “No PNC and institutional delivery” are also confusing. How does one drop out from “No PNC”? I think the authors should clarify with a modified title for Table 2 and a legend for Table 2 to really clarify what these proportions mean. | **Thank you!**   - We try to update the table **( see table 2)** - We consider dropout from institutional delivery as one variable of interest not dropout from the home delivery. - In table 2 we try to describe the dropout rate from PNC from total ANC booked women, institutional delivery, and home delivery. Just to show how the institutional delivery is contributes for the increment of PNC utilization among reproductive age women. |
| 13. Results: Line 152: “We found a high dropout rate from institutional delivery and postnatal care visit after having four and above ANC visit”. Again, it is difficult to read this from Table 2 because I am not sure if the authors are looking at PNC among all – yes/no categories. I am not sure if we are looking at the 15.8% as proportion of those who DID drop out, or as those who HAD postnatal care. The table title says dropout proportion but as stated above, it is presented in a quite ambiguous manner. And the authors should be consistent with the use of proportion vs dropout rate vs dropout proportion. Again, a legend to Table 2 will help. It could help if Table 2 is presented like Table 3, making it clear the dichotomy of drop-out vs no drop-out, compared against the >4 and <4 ANC visit groups. In Table 3, please report the total number from each region. Please refer to the Table numbers in the text when reporting the results, to make it easier for reader to follow. | **Thank you!**   - See table 2. 15.8% was those women who had PNC visit. The dropout rate was 84.2%. - The table three was about dropout from institutional delivery from all booked for ANC, and among those who had recommended number of ANC visit (four and above). And dropout from PNC. We try to update and use proportion instead of rate b/c the data was cross sectional and it is difficult to say rate rather proportion will be appropriate. See the abstract objective, table 2,3 and line 182. |
| 14. Results: Line 164 Being informed or NOT being informed. It seems in 166 the authors state that NOT being informed was associated with drop out. This should be clear in the abstract and the Line 164 to avoid confusion of results. Please do a legend for Table 4 to shed more light on variables such as media exposure and the wealth index. | **Thank you!**   - Acknowledged and it is not being informed about pregnancy complications during their ANC visit. See line 52 of the abstract and line196 of the results. - We make some variables like wealth index and put a legend for them below the table. See table 4 |
| 15. Discussion Line 184-185 might be useful to give one or 2 examples of the rates from these other contexts to make the comparison much clearer. | **Thank you!**   - The dropout rate from maternity continuum of care was high compared to other studies conducted in Nigeria of which women 38.1% and 50.8% of the women who receive ANC were dropped out from skilled delivery and PNC respectively (3), Cambodia (4), and Debremarkos where women had 32.2% dropout from all continuum of care, while 66.4% and 84.1% had four and above ANC, and institutional delivery respectively(5) . see line 217-223 |
| 16. Discussions, Line 185 please specify Debremarkos, Ethiopia since the other two are countries. | **Thank you!**   - The dropout rate from complete continuum of care was 32.2%, 66.4% had 4 and above ANC, 84.1% had institutional delivery. see line 220-221 |
| 17. Discussions: Line 187 what were the rates in Debremarkos? | **Thank you!**   - The dropout rate from complete continuum of care was 32.2%, 66.4% had 4 and above ANC, 84.1% had institutional delivery. see line 220-221 |
| 18. Discussions: Line 189-90 what was the rate? | **Thank you!**   - The magnitude was 10% complete continuum of care, 30.8% used PNC and 29.7% institutional delivery. See the line 227-228 |
| 19. Discussions: Line 194-195 if the authors are to qualify the assertion about decision making, they have to compare some of the contexts in the studies cited that found no significant association with Ethiopia. | **Thank you!**   - The context might be due to variation in the educational status of women, different socio-economic and cultural perspectives where women decision making ability is very important to reduce dropout from maternity continuum of care (6-9). |

1.	Central Statistical Agency (CSA) [Ethiopia] and ICF. Ethiopia Demographic and Health Survey 2016. Addis Ababa, Ethiopia, and Rockville, Maryland, USA: CSA and ICF.

2.	Wudineh KG, Nigusie AA, Gesese SS, Tesu AA, Beyene FY. Postnatal care service utilization and associated factors among women who gave birth in Debretabour town, North West Ethiopia: a community- based cross-sectional study. BMC pregnancy and childbirth. 2018;18(1):508.

3.	Akinyemi JO, Afolabi RF, Awolude OA. Patterns and determinants of dropout from maternity care continuum in Nigeria. BMC pregnancy and childbirth. 2016;16(1):282.

4.	Wang W, Hong R. Levels and determinants of continuum of care for maternal and newborn health in Cambodia-evidence from a population-based survey. BMC pregnancy and childbirth. 2015;15:62.

5.	Amare NS, Araya BM, Asaye MM. Dropout from maternity continuum of care and associated factors among women in Debre Markos town, Northwest Ethiopia. bioRxiv. 2019:620120.

6.	Iqbal S, Maqsood S, Zakar R, Zakar MZ, Fischer F. Continuum of care in maternal, newborn and child health in Pakistan: analysis of trends and determinants from 2006 to 2012. BMC health services research. 2017;17(1):189.

7.	Ryan BL, Krishnan RJ, Terry A, Thind A. Do four or more antenatal care visits increase skilled birth attendant use and institutional delivery in Bangladesh? A propensity-score matched analysis. BMC public health. 2019;19(1):583.

8.	Sakuma S, Yasuoka J, Phongluxa K, Jimba M. Determinants of continuum of care for maternal, newborn, and child health services in rural Khammouane, Lao PDR. PloS one. 2019;14(4):e0215635.

9.	Singh K, Bloom S, Haney E, Olorunsaiye C, Brodish P. Gender equality and childbirth in a health facility: Nigeria and MDG5. African journal of reproductive health. 2012;16(3).
