# Supplementary material for: High dropout rate from maternity continuum of care after antenatal care booking and its associated factors among reproductive age women in Ethiopia, Evidence from Demographic and Health Survey 2016
Source: PLoS One. 2020 Jun 12;15(6):e0234741. doi: 10.1371/journal.pone.0234741 (PMC7292400; doi:10.1371/journal.pone.0234741)
Supplement: Attachment — Submitted filename: Reviewers response version 2 continuum of care.docx [file pone.0234741.s002.docx]

**Author's response to reviews**

High dropout rate from maternity continuum of care after antenatal care booking and its associated factors among reproductive age women in Ethiopia, Evidence from Demographic and Health Survey 2016 (PONE-D-20-01331)

# Atalay Goshu Muluneh*^1^, Getahun Molla Kassa^1^, Geta Asrade Alemayehu^2^, Mehari W/Mariam Merid^1^

*^1^Department of Epidemiology and Biostatistics, Institute of Public Health, College of Medicine and Health Sciences, University of Gondar, Gondar, Ethiopia*

*^2^Department of Health System and Policy, Institute of Public Health, College of Medicine and Health Sciences, University of Gondar, Gondar, Ethiopia*

**Corresponding author:*

Atalay Goshu Muluneh

Email addresses:

AG: [goshuatalay12@gmail.com](mailto:goshuatalay12@gmail.com)

GM: [getahunm08@gmail.com](mailto:getahunm08@gmail.com)

GA: [getasrade64@gmail.com](mailto:getasrade64@gmail.com)

MWM: [mehariho19@gmail.com](mailto:mehariho19@gmail.com)

Postal address: P.O. Box 196, Gondar, Ethiopia

Version 3, Date: May, 2020

With regards!

From: Atalay Goshu Muluneh

Correspondence Author

**Author's response to reviews:** Find it below

To: PLOS ONE, editorial office

**Subject:** Submitting a revised version of a manuscript

**Object:** High dropout from maternity continuum of care after antenatal care booking and its associated factors among reproductive age women in Ethiopia, Evidence from Demographic and Health Survey 2016 (PONE-D-20-01331)

We would like to thank the reviewers and editor for sharing the view and experience. The comments are very important that will improve the manuscript. The point-by-point responses for each of the comments and the revised manuscript are provided in the attached documents.

**POINT BY POINT RESPONSES**

We would like to take this opportunity to thank the reviewers and the editor for sharing their view and constructive comments. The point-by-point responses for each of the comments are provided in the following pages.

Authors’ response of reviewer 1

| **REVIEWER 1 COMMENT** | **AUTHORs RESPONSE** |
| --- | --- |
| 1. Reviewer #1: Most of my previous comments have been addressed. However, I feel the manuscript can still benefit from further language editing. For example, I copied the following section from the Background of the Abstract "Maternal continuums of care are vital for reducing the mortality of mothers and neonates. The proportion of dropouts from the care maintained as a rising phenomenon, while studies on the risk factors associated with defaulters are markedly limited."   The second sentence in the excerpt above is not clear  There are still a couple of sentences such as these throughout which affect smooth readability of the manuscript | Thank you for your suggestion!   - We try to re-read and edit some grammatical errors of the whole document. - The second sentence we are intended to show dropout from maternal continuum of cares remains high but researches related with dropout and associated factors were limited”   - We have modified as “Maternal continuums of care were vital to reducing maternal and neonatal mortalities. While the dropout rate remains high and limited studies were found on risk factors associated with a high dropout rate of the maternal continuum of care” see the background of the Abstract section. |

Reviewer 2

| **REVIEWER COMMENT** | **AUTHORs RESPONSE** |
| --- | --- |
| 1. For instance, the authors say a legend exists for Table 4 as requested, which explain uncommon variables like media exposure (it is not self-explanatory like for instance, age). I am not seeing the legend in revised version. This can actually be one sentence under Table 1 and does not need to repeat for other tables. | *Media exposure:* Media exposure was calculated from the internet use, TV watching, radio listening, reading newspapers and those who score above the median were considered as having media exposure and the rest considered as having no media exposure. See line 155-156 below table 1. |
| 2. Line 170-171 still needs to be fixed like how the authors addressed it in the comment to reviewer. It is still unclear in the manuscript. This is the comment to the reviewer "Among women who booked for ANC only 308 (6.56%) used the complete continuum of care. Among women who had a recommended ANC (four and above) 11.84% used the complete continuum of care." This is much clearer than the way it is currently put in the manuscript | Thank you!   - We accepted and incorporate the reviewers comment. See line 157-159 |
| 3. Line 219-221 it talks about proportions for both Debremarkos and Cambodia - these two places share the same statistics? | Thank you very much.   - We acknowledge and the magnitude of dropout from continuum of care in Cambodia was missed. About 90 % had at least one ANC, 60% of them 4 and above, 74 % Skill birth attendance and 71% had at least one postnatal checkup. We incorporate it from the main manuscript. See line 203-205 |

Answers for Editors’ comment/question

| **Editor COMMENT** | **AUTHORs RESPONSE** |
| --- | --- |
| 1. Please amend the title either on the online submission form or in your manuscript so that they are identical | We amended at as the online submitted one and see line 1-3 of the manuscript |
| 4. You have indicated that data is available from [http://www.dhsprogram.com](http://www.dhsprogram.com/). Before we proceed with your manuscript, please address the following issues in your Data Availability Statement:  a) Please provide a direct link to the database(s) used in your study, or name the specific database(s) used.  b) Please remove "The authors prepared the data that was used for preparation of this manuscript can be shared if required."  Your manuscript has been returned to your account. Please log on to PLOS Editorial Manager at <https://www.editorialmanager.com/pone/> to access your manuscript. | Thank you!   - We remove the sentence” The authors prepared the data that was used for preparation of this manuscript can be shared if required” - We write the name of the specific data base “We used the Ethiopian Demographic and Health Survey 2016 women data” but specific link is not available for this data base. Anyone could register as an authorized user from the major Demographic and Health Survey website <http://www.dhsprogram.com> and could request specific data set of any countries. See line 279-281 of the manuscript. |
| 5. Please confirm whether the following Data Availability Statement looks acceptable.  "The data used for the preparation of this manuscript were taken from the "Ethiopian Demographic and Health Survey 2016". Users can access the data at [http://www.dhsprogram.com](http://www.dhsprogram.com/)." | Thank you,  Accepted and corrected accordingly. See line 279-280 |
